# The prediction value of PI-RADS v2 score in high-grade Prostate Cancer: a multicenter retrospective study

**DOI:** 10.7150/ijms.45730

**Published:** 2020-05-30

**Authors:** Song Chen, Yun Yang, Tianchen Peng, Xi Yu, Haiqing Deng, Zhongqiang Guo

**Affiliations:** 1 Department of Urology, Zhongnan Hospital of Wuhan University, Wuhan, 430071, China.; 2 Department of Dermatology, The First Affiliated Hospital of Harbin Medical University, Harbin, 150001, China.; 3 Department of Urology, Renmin Hospital of Wuhan University, Wuhan, 430060, China.; 4 Department of Urology, Xiangyang Central Hospital, Xiangyang, 441021, China.

**Keywords:** high-grade prostate cancer, PI-RADS v2, nomogram, prediction model

## Abstract

**Background:** To explore the prediction value of PI-RADS v2 in high-grade prostate cancer and establish a prediction model combined with related variables of prostate cancer.

**Material and Methods:** A total of 316 patients with newly discovered prostate cancer at Zhongnan Hospital of Wuhan University and Renmin Hospital of Wuhan University from December 2017 to August 2019 were enrolled in this study. The clinic information as age, tPSA, fPSA, prostate volume, Gleason score and PI-RADS v2 score have been collected. Univariate analysis was performed based on every variable to investigate the risk factors of high-grade prostate cancer. ROC curves were generated for the risk factors to distinguish the cut-off points. Logistic regression analyses were used to investigate the independent risk factors of high-grade prostate cancer. Nomogram prediction model was generated based on multivariate logistic regression analysis. The calibration curve, ROC curve, leave-one-out cross validation and independent external validation were performed to evaluate the discriminative ability, accuracy and stability of the nomogram prediction model.

**Results:** Of 316 patients, a total of 187 patients were diagnosed as high-grade prostate cancer. Univariate analysis showed tPSA, fPSA, prostate volume, PSAD and PI-RADS v2 score were significantly different between the high- and low-grade prostate cancer patients. Univariate and multivariate logistic regression analyses showed only tPSA, prostate volume and PI-RADS v2 score were the independent risk factors of high-grade prostate cancer. The nomogram could predict the probability of high-grade prostate cancer, with a sensitivity of 79.4% and a specificity of 77.6%. The calibration curve displayed good agreement of the predicted probability with the actual observed probability. AUC of the ROC curve was 0.840 (0.797-0.884). Leave-one-out cross validation indicated the nomogram prediction model could classify 81.4% cases accurately. External data validation was performed with a sensitivity of 80.6% and a specificity of 77.3%, the Kappa value was 0.5755.

**Conclusions:** PI-RADS v2 score had the value in predicting high-grade prostate cancer and the nomogram prediction model may help early diagnose the high risk prostate cancer.

## Introduction

Prostate cancer (PCa) is the second most common malignancy in male worldwide and the second leading cause of cancer death in male 1. In recent years, the incidence and mortality of prostate tumor in China have increased year by year, and the growth rate ranks first among male malignancies 2.

PCa is usually classified as low-grade tumor and high-grade tumor. Most studies considered Gleason score (GS) ≤3+3 as a low-grade PCa, GS ≥7 as a high-grade PCa, but in recent years, more and more evidence indicated that the metastasis probability, 10-years cancer specific survival and biochemical recurrence after radical resection of PCa patients with a GS =3+4 were closer to patients with a GS =3+3 3-5. The treatment and prognosis of low- and high-grade prostate cancers are evidently different 6. American Urological Association (AUA) Guideline recommended biopsy was not needed for low-grade PCa patients, only dynamic follow-up 7. Similarly, Chinese Urological Association (CUA) Guideline also recommended dynamic monitor for PCa patients with a GS ≤3+4, which could avoid erectile dysfunction, urinary incontinence and other complications after radical prostatectomy, and improve the life quality of patients; while PCa patients with a GS ≥4+3 should adopt more active treatment strategies, such as radical prostatectomy, endocrine therapy or radiotherapy and chemotherapy, so as to prolong the survival time of patients 8. It is of great significance to identify low- and high-grade prostate cancers before treatment.

At present, more and more attention is paid on multi-parameter prostate magnetic resonance imaging (MRI), which is the main method of imaging diagnosis of prostate cancer. The Prostate Imaging Reporting and Data System version 1 (PI-RADS v1) was published by European Society of Urogenital Radiology (ESUR) in 2012 and developed into the PI-RADS v2 in 2015 9. The PI-RADS v2 was mainly aimed at improving detection, localization, characterization, the risk stratification in patients with suspected tumor and the standardization of diagnosis and reporting of high-grade PCa 10. Some studies indicated the PI-RADS v2 had good value in diagnosing clinically significant PCa 10-12. Our previous study also demonstrated the predictive value of PI-RADS v2 score in PCa bone metastasis 13. So far, several studies have confirmed age, PSA, prostate volume, PSA density (PSAD), abnormal digital rectal examination (DRE) may be the factors influencing high-grade PCa 14-17. In addition, many prediction models have been established for high-grade PCa in foreign country, which were mainly based on PSA and derived parameters, few models incorporating multiparametric prostate MRI such as PI-RADS v2 score 18-20. Furthermore, due to the differences in races and morbidity, it needs further confirming whether these models are appropriate for Chinese patients, and most of these studies are lack of external data validation.

Thus, we conducted a multicenter retrospective study to determine the prediction value of the PI-RADS v2 score in high-grade PCa and establish a prediction model combined with related variables of prostate cancer.

## Material and methods

### Study patients

The study patients consisted of a development cohort and a validation cohort. The development cohort included 316 patients with newly discovered PCa at Zhongnan Hospital of Wuhan University and Renmin Hospital of Wuhan University from December 2017 to August 2019. The clinic information as age, tPSA, fPSA, prostate volume, Gleason score and PI-RADS v2 score were collected. The pathological result of ultrasound guided prostate biopsy or radical prostatectomy was as the outcome variable. In this study, we considered GS ≤3+4 as a low-grade PCa, GS ≥3+4 as a high-grade PCa. We retrospectively reviewed medical records of all enrolled patients to acquire the clinical information. An independent cohort included 53 patients from Xiangyang Central Hospital (January 2018 to October 2019) was used to validate the nomogram prediction model. All patients provided the informed consent. The Ethics Committee at Zhongnan Hospital of Wuhan University had approved the using clinical information in our study (approval number: 2015029). All procedures and ethical standards were done in accordance with the national research committee and/or institutional.

### Inclusion criteria

Patients were enrolled in this study if they met all the following criteria: (i) the prostate cancer patients; (ii) patients who underwent ultrasound guided prostate biopsy or radical prostatectomy; (iii) patients who underwent multiparameter MRI of the prostate (T2 WI, DWI, DCE imaging), and prostate multiparameter MRI distanced biopsy or radical prostatectomy time was within one month; (iv) had complete and detailed clinical, pathological data record.

### Exclusion criteria

Patients meeting any of the following criteria were excluded: (i) merge other tumors; (ii) patients had received treatment before multiparameter MRI examination, such as hormone therapy, radiotherapy; (iii) any incomplete clinical or pathological data.

### Statistical analysis

Age, fPSA and PSAD were analyzed by two-sample t test and graded variables were analyzed with Mann-Whitney test or Chi-square test. Univariate analysis was performed based on every variable to investigate the risk factors of high-grade PCa. Receiver operating characteristic (ROC) curves were generated for the risk factors to distinguish the cut-off points and the areas under the curves (AUCs) were compared. Univariate and multivariate logistic regression analyses were used to investigate the independent risk factors of high-grade PCa. Nomogram prediction model was generated based on multivariate logistic regression analysis. The calibration curve was generated to assess the agreement of the nomogram-predicted probability with the actual observed probability. We estimated the prediction error of the nomogram prediction model using leave-one-out cross validation, the detail method information was as described by Simon et al 21. ROC curve, leave-one-out cross validation and independent external validation were performed to evaluate the discriminative ability, accuracy and stability of the nomogram prediction model. We used SPSS 16.0 to perform all statistical analyses. Nomogram and calibration curve were generated with R version 3.5.0 and a p value <0.05 was considered statistically significant.

## Results

### Patient characteristics and univariate analysis for prostate cancer

The detailed clinical parameters of development cohort were displayed in Table 1, no significant difference was observed in clinical parameters between the two hospitals (all p>0.05).

In the development cohort, 187 (59.2%) of 316 patients were classified as high-grade PCa. The mean age was 73.1 ± 8.5 years and the median age was 73 years. The mean age of high-grade PCa patients was 73.5 ± 8.1 years, the median age was 74 years, and the mean age of low-grade PCa patients was 72.6 ± 7.9 years, with a median age of 72 years. Two-sample t test showed that only fPSA and PSAD were significantly different between the high- and low-grade prostate cancer patients (p<0.05). Mann-Whitney test and Chi-square indicated that tPSA, prostate volume and PI-RADS v2 were significantly different between the two groups (p<0.05). The age and fPSA/tPSA had no statistical difference between two groups (Table 2).

### ROC curves were generated for the risk factors to distinguish the cut-off points

**Figure 1:**
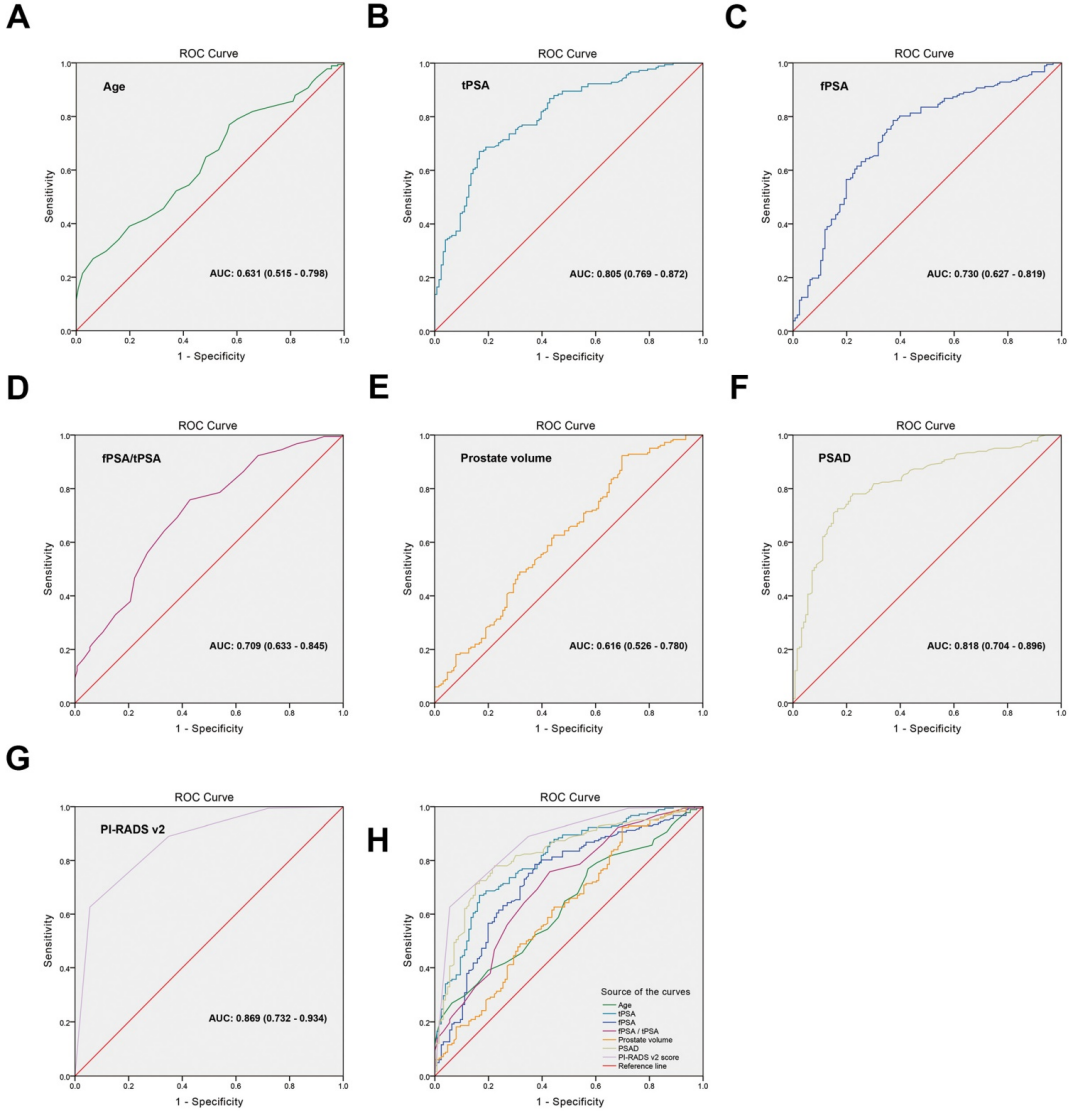
** ROC curves were generated for the risk factors to distinguish the cut-off points. (A)** age, AUC: 0.631 (0.515-0.798), **(B)** tPSA, AUC: 0.805 (0.769-0.872), **(C)** fPSA, AUC: 0.730 (0.627-0.819), **(D)** fPSA/tPSA, AUC: 0.709 (0.633-0.845),** (E)** prostate volume, AUC: 0.616 (0.526-0.780),** (F)** PSAD, AUC: 0.818 (0.704-0.896), **(G)** PI-RADS v2 score, AUC: 0.869 (0.732-0.934), **(H)** Comparison of the ROC curve for each variable.

To distinguish the cut-off points of high-grade PCa risk factors, ROC curves were generated and the AUCs were compared. The cut-off point of every variable was set based on the value of the maximum sum of the sensitivity and specificity on the ROC curve. Figure 1 and Table 3 showed that the AUCs were 0.631 (95% CI: 0.515-0.798), p=0.171) for age, 0.805 (95% CI: 0.769-0.872, p=0.028) for tPSA, 0.730 (95% CI: 0.627-0.819, p=0.042) for fPSA, 0.709 (95% CI: 0.633-0.845, p=0.057) for fPSA/tPSA, 0.616 (95% CI: 0.526-0.780, p=0.006) for prostate volume, 0.818 (95% CI: 0.704-0.896, p=0.344) for PSAD, and 0.869 (95% CI: 0.732-0.954, p<0.001) for PI-RADS v2 score. The cut-off points of high-grade prostate cancer risk factors were as follows: age was ≥68 years, tPSA was ≥16.47 ng/mL, fPSA was ≥4.56 ng/mL, fPSA/tPSA was ≤0.08, prostate volume was ≤64.4 cm^3^, PSAD was ≥0.61 ng/mL/cm^3^, PI-RADS v2 score was ≥4. The Youden index, sensitivity and specificity of every variable were listed in Table 3.

### Univariate and multivariate logistic regression analyses

Univariate logistic regression analysis showed that age was not the risk factors of high-grade PCa (p>0.05), whereas tPSA, fPSA, fPSA/tPSA, prostate volume, PSAD and PI-RADS v2 score were the risk factors (p<0.05). The OR values were as follows: PI-RADS v2 score (OR=3.751), PSAD (OR=2.496), fPSA/tPSA (OR=0.448), tPSA (OR=1.264), fPSA (OR=1.172), prostate volume (OR=0.935). Furthermore, multivariate logistic regression analysis showed that only tPSA (OR=1.428, p=0.029), prostate volume (OR=0.943, p=0.041) and PI-RADS v2 score (OR=2.162, p=0.002) were the independent risk factors of high-grade PCa (Table 4).

### Construction of nomogram and calibration curve to predict high-grade prostate cancer

**Figure 2:**
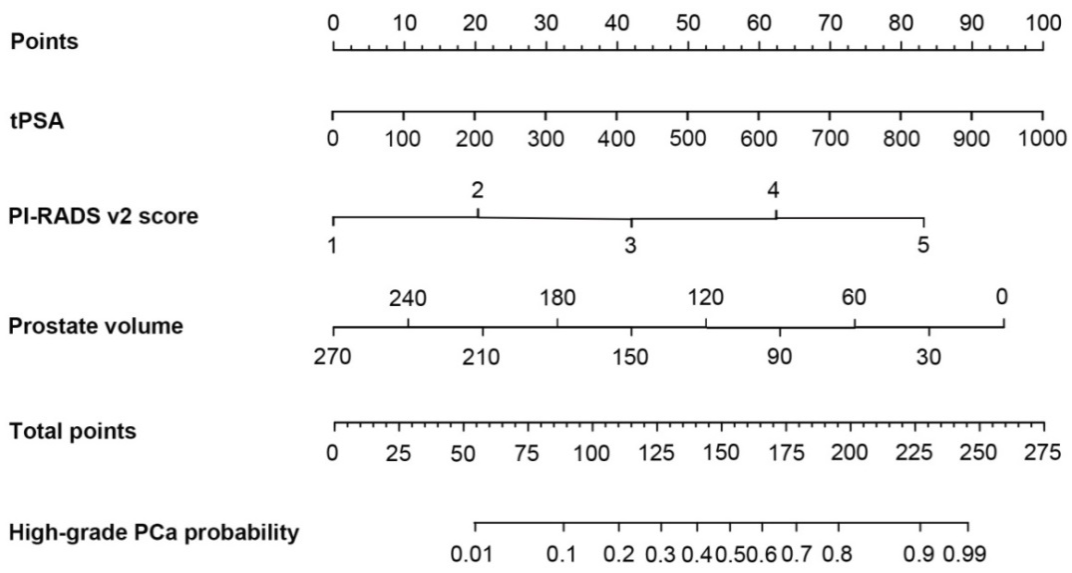
** The nomogram was developed for high-grade prostate cancer.** To estimate the risk of high-grade prostate cancer, the points for each variable were calculated by drawing a straight line from a patient's variable value to the axis labelled “Points”. The score sum is converted to a probability in the lowest axis.

**Figure 3:**
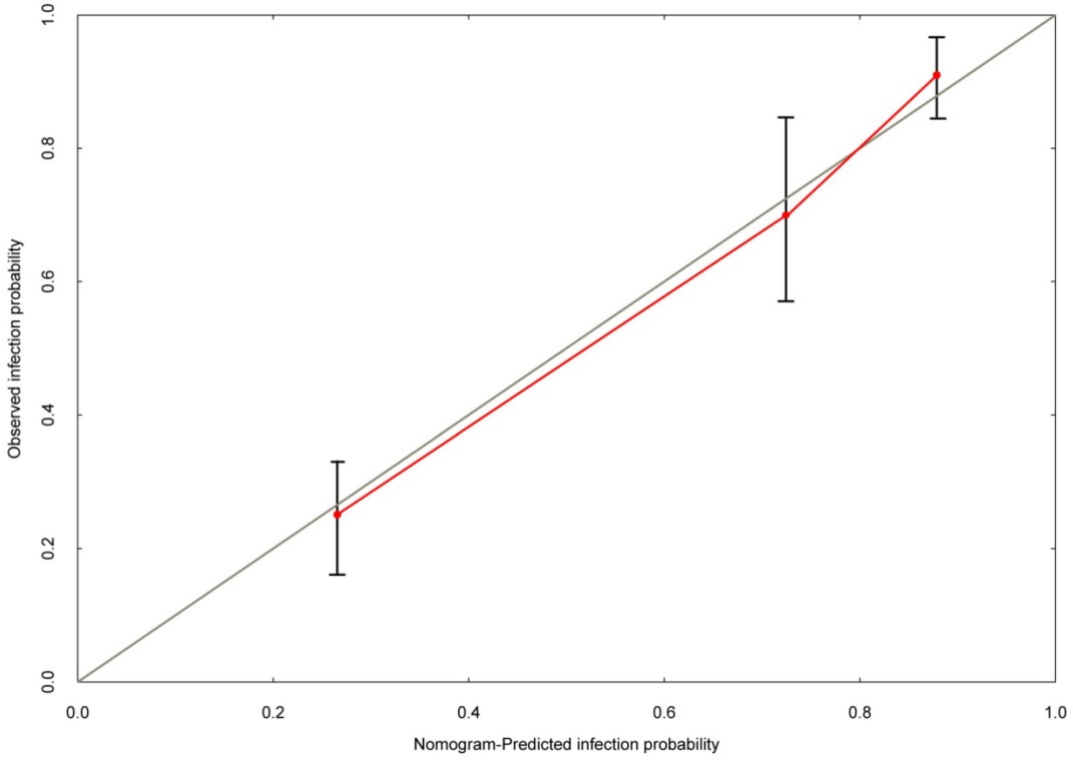
** The calibration curve was developed for high-grade prostate cancer.** The nomogram-predicted probability is plotted on the x-axis, and the actual probability is plotted on the y-axis.

Based on the multivariate logistic regression analysis, tPSA, prostate volume and PI-RADS v2 score could be enrolled to generate the nomogram and calibration curve to predict high-grade PCa. Corresponding to each variable on the nomogram (Figure 2), the total score was calculated to predict the probability of infection in each patient. In the nomogram, the scores corresponding to the vertical line on the “score” ruler by all the variable values of the patient were found, accumulated the scores of all the variable values and found the vertical line of the “predictive ruler” on the accumulated “total score” ruler. The corresponding point was converted to the corresponding probability on the “High-grade PCa probability” scale according to the score on the predicted ruler, which was the probability of patient with high-grade PCa. The clinical information of each patient was included in the nomogram for matching analysis. The sensitivity was 79.4% and the specificity was 77.6%. The calibration curve (Figure 3) displayed good agreement of the predicted probability with the actual observed probability for high-grade PCa, which indicated that the nomogram had good accuracy.

### Evaluation of the nomogram prediction model for high-grade prostate cancer

**Figure 4:**
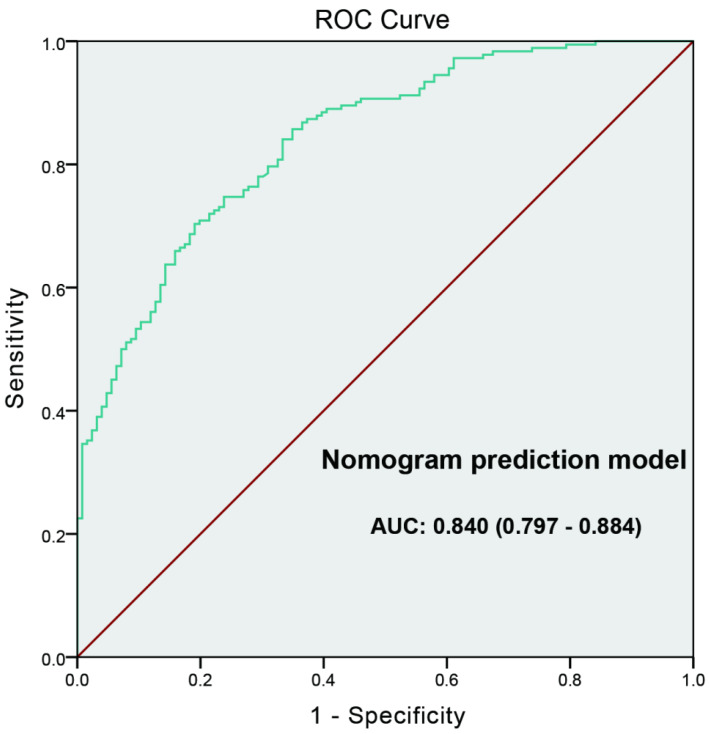
** The ROC curve developed for nomogram prediction model of high-grade prostate cancer.** The AUC of prediction model was 0.840 (0.797-0.884).

ROC curve was generated to evaluate the value of the nomogram prediction model; the “high-grade PCa” was as the outcome variable (Figure 4). The AUC of ROC curve was 0.840 (0.797-0.884). Leave-one-out cross validation indicated the nomogram prediction model could classify 81.4% cases accurately. It was been proved again that the nomogram prediction model had good discriminative ability and accuracy. To confirm the stability of the model, external data validation was performed, which was independently collected in Xiangyang Central Hospital. The sensitivity was 80.6% and the specificity was 77.3%, the Kappa value was 0.5755 (Table 5).

## Discussion

Pathological grade of PCa is closely related to treatment and prognosis. Some patients with high-grade PCa usually have distant metastasis, such as bone metastasis, lung metastasis. Different from non-metastatic PCa, radical prostatectomy is no longer the main treatment. In a large sample retrospective analysis, Albertsen et al. found when the Gleason score was 8-10, patients had an obviously higher 10-year mortality rate (12.1%) compared with low-grade PCa 22. Therefore, it is necessary to study how to improve the diagnostic accuracy of high-grade PCa.

Most studies considered GS ≤3+3 as a low-grade PCa, GS ≥7 as a high-grade PCa, but in recent years, more and more evidence indicated that the metastasis probability, 10-years cancer specific survival and biochemical recurrence after radical resection of PCa patients with a GS=3+4 were closer to patients with a GS=3+3 3-5. CUA Guideline (2014 edition), European Association of Urology (EAU) Guideline (2016 edition) and European Society for Medical Oncology (ESMO) Guideline (2015 edition) stated that PCa patients with a GS=4+3 had remarkably different prognosis from who with a GS=3+4, and recommended different interventions 8,23-24. Hence, we considered GS ≤3+4 as a low-grade PCa and GS ≥3+4 as a high-grade PCa in this study.

The detailed clinical parameters of enrolled patients in development cohort from the two hospitals had no significant difference, demonstrating the universality of the enrolled patients. Univariate analysis showed tPSA, fPSA, prostate volume, PSAD and PI-RADS v2 were significantly different between the high-grade PCa patients and low-grade PCa patients, mainly consistent with previous studies 14-17. We generated ROC curves and found that age, fPSA, fPSA/tPSA and prostate volume were not ideal diagnostic parameters because of low sensitivity (<70%) or low specificity (<70%). Park et al. demonstrated that PI-RADS v2 score could help preoperatively predict clinically significant prostate cancers, with the AUC was about 0.80, although it was higher than AUC of tPSA, there was no statistical difference between them 10. The results were basically consistent with our study. PSAD refers to the PSA content of a prostate per unit volume. PSAD was reported to significantly increase tumor detection rate and had a closely relationship with tumor invasiveness 25. The results in our study showed that AUC of PI-RADS v2 score was the highest (0.869), and PSAD was the second (0.818). The cut-off point of PI-RADS v2 score was ≥4, as same as our previous study 13. But the cut-off point of PSAD was ≥0.61 ng/mL/cm^3^, higher than 0.15-0.35 in previous studies 15-17. The analysis of the reasons may be as follows: (i) the scope of tPSA in this study was large (1.57-964.43 ng/ml), not limited to 4-10 ng/ml; (ii) mainly for high-grade tumors, Li et al. 26 found that Gleason score <7 group, PSAD average ± standard deviation was (0.43±0.48) ng/ml/cm^3^, and Gleason score ≥7 group, PSAD average ± standard deviation was (2.55±11.06) ng/ml/cm^3^, the difference p value between the two groups was <0.001. Therefore, it was reasonable to believe that when the research object was high-grade prostate cancer, the cut-off point of PSAD will increased.

In this study, univariate logistic regression analysis showed tPSA, fPSA, fPSA/tPSA, prostate volume, PSAD and PI-RADS v2 score were the risk factors (p<0.05). Furthermore, multivariate logistic regression analysis showed that only tPSA (OR=1.428, p=0.029), prostate volume (OR=0.943, p=0.041) and PI-RADS v2 score (OR=2.162, p=0.002) were the independent risk factors of high-grade PCa.

Several reports in domestic and foreign have declared that nomogram had been established to predict PCa through prostate related parameters such as PSA 27-29. Stamatakis et al. reported that nomogram based on magnetic resonance multi-parameter imaging could be used to screen patients for dynamic follow-up, and prostate multi-parameter MRI results played an important role in clinical decision-making 30. Tang et al. 31 established a nomogram based on PSA, prostate volume and DRE for predicting Chinese prostate cancer. The AUC of the nomogram was 0.848, which was higher than PSA alone. Based on the age, PSA and its derived parameters, DRE and ultrasound examination results, Huang et al. 32 constructed a nomogram that reduces the number of prostate puncture biopsy needles. The AUC was 0.853, higher than PSA alone (AUC=0.761).

Based on the multivariate logistic regression analysis, we constructed nomogram and calibration curve to forecast the probability of high-grade PCa, including tPSA, prostate volume and PI-RADS v2 score. The sensitivity was 79.4% and the specificity was 77.6%. The calibration curve displayed good agreement of the predicted probability with the actual observed probability for high-grade PCa, which indicated that the nomogram had good accuracy. In addition, ROC curve was generated to evaluate the value of the nomogram prediction model, with the AUC was 0.840 (0.797-0.884). Leave-one-out cross validation indicated the nomogram prediction model could classify 81.4% cases accurately. It was been proved again that the nomogram prediction model had good discriminative ability and accuracy. To confirm the stability of the model, external data validation was performed, which was independently collected in Xiangyang Central Hospital. The sensitivity was 80.6% and the specificity was 77.3%, the kappa value was 0.5755.

Taken together, the results showed that the nomogram prediction model exhibited good discriminative ability, accuracy and stability. It meant the prediction model had great value of prediction and could be well generalized for other independent datasets.

Unavoidable, some shortcomings in this study need to state. This study is a retrospective study, and there are inevitably biases, such as selection bias. Besides, the size of the study sample is small and it is subject to further study by expanding the sample. Moreover, although the verification of the results was relatively adequate and the cross validation, external data validation were performed verifying the discriminative ability, accuracy and stability of the nomogram, a prospective study is needed to further confirm the reliability of the results.

## Conclusion

Based on 316 prostate cancer patients from two hospitals, this study evaluated the risk factors of high-grade PCa, indicating that PI-RADS v2 score, tPSA and prostate volume were the independent risk factors of high-grade PCa. Moreover, based on the multivariate logistic regression analysis, we established a nomogram as prediction model to calculate the probability of high-grade PCa, calibration curve, ROC curve, cross validation and external validation displayed that the nomograms had great value of prediction. The nomogram prediction model may help early diagnose the high risk prostate cancer.

## Figures and Tables

**Table 1:** Clinical characteristics of prostate cancer patients in two hospitals

Variables	All patients (n=316)	Zhongnan Hospital (n=257)	Renmin Hospital (n=59)	*p* value
**Age/years**				0.818
Average/Median (Range)	73.1±8.5/73	73.0±8.2/73	73.4±7.9/73	
	46-90	46-90	51-89	
**tPSA (ng/mL), n (%)**				0.176
Average/Median (Range)	139.57±195.68/51.42	143.93±184.56/52.85	118.42±155.70/48.38	
	1.57-964.43	1.57-964.43	4.08-862.26	
≤10.0	38 (12.0)	30 (11.6)	8 (13.6)	
10.1-20.0	58 (18.4)	45 (17.5)	13 (22.0)	
20.1-100.0	113 (35.8)	94 (36.6)	19 (32.2)	
>100.0	107 (33.9)	88 (34.2)	19 (32.2)	
**fPSA (ng/mL)**				0.252
Average/Median (Range)	11.51±12.16/9.45	12.08±11.54/10.02	9.17±8.63/8.15	
	0.13-69.16	0.13-69.16	0.21-54.33	
**Prostate volume (cm^3^), n (%)**				0.439
Average/Median (Range)	46.15±30.83/43.98	47.06±29.11/44.25	44.38±20.42/40.78	
	11.93-261.52	12.24-261.52	11.93-196.40	
≤30	112 (35.4)	88 (34.2)	24 (40.7)	
30.1-60	125 (39.6)	100 (38.9)	25 (42.4)	
60.1-90	52 (16.5)	45 (17.5)	7 (11.9)	
>90	27 (8.5)	24 (9.3)	3 (5.1)	
**PSAD (ng/mL/cm^3^)**				0.901
Average/Median (Range)	0.62±0.54/0.63	0.63±0.51/0.63	0.60±0.46/0.60	
	0.25-4.69	0.25-4.69	0.26-4.63	
**fPSA/tPSA, n (%)**				0.944
Average/Median (Range)	0.12±0.09/0.11	0.12±0.08/0.11	0.12±0.07/0.12	
	0.04-0.61	0.04-0.61	0.06-0.57	
≤0.16	227 (71.8)	182 (70.8)	45 (76.3)	
>0.16	89 (28.2)	75 (29.2)	14 (23.7)	
**Gleason score, n (%)**				0.385
≤6	86 (27.2)	67 (26.0)	19 (32.2)	
3+4	43 (13.6)	37 (14.4)	6 (10.2)	
4+3	50 (15.8)	41 (16.0)	9 (15.3)	
8-10	137 (43.4)	112 (43.6)	25 (42.4)	
**PI-RADS v2 score, n (%)**				0.797
1-2	10 (3.2)	8 (3.1)	2 (3.4)	
3	71 (22.5)	59 (23.0)	12 (20.3)	
4	124 (39.2)	100 (38.9)	24 (40.7)	
5	111 (35.1)	90 (35.0)	21 (35.6)	

**Table 2:** Univariate analysis for high-grade prostate cancer and low-grade prostate cancer

Variables	Low-grade prostate cancer (n=129)	High-grade prostate cancer (n=187)	*p* value
**Age/years**			0.350
Average/Median (Range)	72.6±7.9/72	73.5±8.1/74	
	46-85	51-90	
**tPSA (ng/mL), n (%)**			0.029
Average/Median (Range)	87.04±112.65/32.70	161.19±184.36/68.57	
	1.57-364.43	4.21-964.43	
≤10.0	23 (17.8)	15 (8.0)	
10.1-20.0	34 (26.4)	24 (12.8)	
20.1-100.0	43 (33.3)	70 (37.4)	
>100.0	29 (22.5)	78 (41.7)	
**fPSA (ng/mL)**			0.013
Average/Median (Range)	5.26±7.31/4.18	15.89±10.40/12.95	
	0.13-17.16	1.64-69.16	
**Prostate volume (cm^3^), n (%)**			0.048
Average/Median (Range)	52.31±25.92/46.35	43.06±23.14/39.78	
	18.50-261.52	11.93-204.83	
≤30	42 (32.6)	70 (37.4)	
30.1-60	49 (38.0)	76 (40.6)	
60.1-90	23 (17.8)	29 (15.5)	
>90	15 (11.6)	12 (6.4)	
**PSAD (ng/mL/cm^3^)**			0.041
Average/Median (Range)	0.57±0.48/0.58	0.66±0.51/0.67	
	0.25-4.02	0.28-4.69	
**fPSA/tPSA, n (%)**			0.764
Average/Median (Range)	0.13±0.07/0.12	0.12±0.08/0.11	
	0.05-0.61	0.04-0.60	
≤0.16	91 (70.5)	136 (72.7)	
>0.16	38 (29.5)	51 (27.3)	
**PI-RADS v2 score, n (%)**			<0.001
1-2	8 (6.2)	2 (1.1)	
3	43 (33.3)	28 (15.0)	
4	44 (34.1)	80 (42.8)	
5	34 (26.4)	77 (41.2)	

**Table 3:** The diagnostic value of each variable in high-grade prostate cancer

Variables	Cut-off point	Youden index	AUC (95% CI)	Sensitivity	Specificity	*p* value
Age (years)	≥68	0.28	0.631 (0.515-0.798)	0.77	0.54	0.171
tPSA (ng/mL)	≥16.47	0.57	0.805 (0.769-0.872)	0.81	0.72	0.028
fPSA (ng/mL)	≥4.56	0.46	0.730 (0.627-0.819)	0.61	0.75	0.042
fPSA/tPSA	≤0.08	0.43	0.709 (0.633-0.845)	0.63	0.71	0.057
Prostate volume (cm^3^)	≤64.4	0.22	0.616 (0.526-0.780)	0.64	0.60	0.006
PSAD (ng/mL/cm^3^)	≥0.61	0.60	0.818 (0.704-0.896)	0.70	0.85	0.344
PI-RADS v2 score	≥4	0.67	0.869 (0.732-0.934)	0.73	0.88	<0.001

**Table 4:** Univariate and multivariate logistic regression analyses for high-grade prostate cancer

Variables	Univariate analysis			Multivariate analysis		
	OR	95% CI	*p* value	OR	95% CI	*p* value
Age (years)	1.033	0.945-1.168	0.289	1.026	0.958-1.187	0.536
tPSA (ng/mL)	1.264	1.120-1.439	0.045	1.428	1.175-1.764	0.029
fPSA (ng/mL)	1.172	1.086-1.253	0.034	1.051	0.840-1.263	0.718
fPSA/tPSA	0.448	0.253-0.826	0.007	0.815	0.641-1.076	0.547
Prostate volume (cm^3^)	0.935	0.864-0.981	0.023	0.943	0.912-0.990	0.041
PSAD (ng/mL/cm^3^)	2.496	1.787-3.642	0.041	0.967	0.846-2.179	0.983
PI-RADS v2 score	3.751	2.069-5.380	<0.001	2.162	1.473-3.548	0.002

**Table 5:** External data validation of the prediction model for high-grade prostate cancer

		High-grade prostate cancer (Validation cohort)		Total
		No	Yes	
High-grade prostate cancer (Prediction model)	No	17	5	22
	Yes	6	25	31
Total		23	30	53

Sensitivity = 25/(25+6)=80.6% Specificity = 17/(17+5)=77.3% Kappa value = 0.5755

## Abbreviations

AUA: American Urological Association
AUCs: Areas Under the Curves
CUA: Chinese Urological Association
DRE: Digital Rectal Examination
EAU: European Association of Urology
ESMO: European Society for Medical Oncology
ESUR: European Society of Urogenital Radiology
GS: Gleason Score
MRI: Magnetic Resonance Imaging
PCa: Prostate Cancer
PI-RADS: Prostate Imaging Reporting and Data System
ROC: Receiver Operating Characteristic
PSAD: Prostate Specific Antigen Density
